# Comparable outcomes with low-dose and standard-dose horse anti-thymocyte globulin in the treatment of severe aplastic anemia

**DOI:** 10.1007/s44313-024-00003-z

**Published:** 2024-02-26

**Authors:** Arihant Jain, Aditya Jandial, Thenmozhi Mani, Kamal Kishore, Charanpreet Singh, Deepesh Lad, Gaurav Prakash, Alka Khadwal, Reena Das, Neelam Varma, Subhash Varma, Pankaj Malhotra

**Affiliations:** 1 Department of Clinical Hematology and Medical Oncology, PGIMER, Chandigarh, 160012 India; 2 Department of Biostatistics, CMC, Vellore, Hematology India; 3 Department of Biostatistics, PGIMER, Chandigarh, India; 4 Department of Hematology, PGIMER, Chandigarh, India

**Keywords:** Horse anti-thymocyte globulin, Dose, Aplastic anemia, Response

## Abstract

**Background:**

The standard dose (SD) of horse anti-thymocyte globulin (hATG) ATGAM (Pfizer, USA) or its biosimilar thymogam (Bharat Serum, India) for the treatment of Aplastic Anemia (AA) is 40 mg/kg/day for 4 days in combination with cyclosporine. Data on the impact of hATG dose on long-term outcomes are limited. Here, we describe our comparative experience using 25 mg/kg/day (low-dose [LD]) hATG for 4 days with SD for the treatment of AA.

**Methods:**

We retrospectively studied patients with AA (age > 12 years) who received two doses of hATG combined with cyclosporine. Among 93 AA patients who received hATG, 62 (66.7%) and 31 (33.3%) patients received LD and SD hATG with cyclosporine, respectively. Among these,seventeen(18.2%) patients also received eltrombopag with hATG and cyclosporine. Overall response rates [complete response (CR) and partial response (PR)] of LD and SD hATG groups at 3 months (50% vs. 48.4%; *p* = 0.88), 6 months (63.8% vs. 71.4%; *p* = 0.67), and 12 months (69.6% vs. 79.2%; *p* = 0.167) were comparable. The mean (Standard Deviation) 5-year Kaplan–Meier estimate of overall survival and event-free survival was 82.1 (4.6)% and 70.9 (5.5)% for the study population. The mean (standard deviation) 5-year Kaplan–Meier estimate of overall survival and event-free survival of those who received LD hATG versus SD hATG dose was 82.9 (5·3)% versus 74.8 (10·3)% (*P* = 0·439), and 75.2 (6.2)% versus 61.4(11.2)% (*P* = 0·441).

**Conclusion:**

Our study revealed that the response rates of patients with AA and LD were similar to those of patients with SD to hATG combined with cyclosporine in a real-world setting.

**Supplementary Information:**

The online version contains supplementary material available at 10.1007/s44313-024-00003-z.

## Introduction

Acquired aplastic anemia (AA) is a life-threatening hematological disorder characterized by cytopenias of multiple cell lines. In the presence of a suitable donor, allogeneic hematopoietic cell transplantation (HCT) is the treatment of choice with excellent outcomes [1–4]. However, allogeneic HSCT may not be feasible for a significant proportion of patients owing to a lack of suitable donors, fitness, or resource constraints [5, 6]. In such cases, immunosuppressive therapy (IST) with horse anti-thymocyte globulin (hATG) in combination with cyclosporine has been the mainstay of management [7, 8]. Recently, the addition of thrombopoietin receptor agonists has further improved the efficacy of IST with hATG and cyclosporine [9, 10].

ATGAM (Pfizer, USA), Lymphoglobuline® (Genzyme, Cambridge, MA, USA), and Thymoglobuline® (Genzyme, Cambridge, MA, USA) are the most frequently used ATG preparations for AA. Thymogam (Bharat Serums and Vaccines, India) is a generic version of ATGAM that has been used for more than a decade. ATGAM, lymphoglobulin, and Thymogam are horse ATG (hATG) preparations, whereas thymoglobulin is a rabbit ATG (rATG) preparation. Although randomized trials have shown the superiority of hATG over rATG in the frontline treatment of AA, studies on the impact of ATG dose on long-term outcomes are limited [11]. The dose and timing of ATG are likely to affect the degree of immune modulation, hematologic response, infection risk, and cost of treatment [12]. In the setting of allogeneic HSCT, a reduction in the rATG dose as part of the conditioning regimen did not translate into inferior outcomes in most studies [13–16].

The higher incidence of AA in low-middle-income countries (LMICs) has been linked to socioeconomic status [17, 18]. Moreover, financial constraints continue to be one of the major deterrents in access to IST among AA patients in LMICs where a majority of patients lack access to health insurance and spend money out-of-pocket to meet treatment expenses [18]. In India, IST with cyclosporine and standard-dose hATG (40 mg/kg/day × 4 days) costs approximately 6000–9000 USD for an average adult patient with AA weighing 60 kg. We previously reported comparable short-term efficacy of low-dose hATG (25 mg/kg/d × 4 days) and standard-dose hATG (40 mg/kg/d × 4 days) [19]. hATG has also been used at lower doses in elderly patients with AA [20]. Long-term comparative data on the outcomes of the two dosing regimens are scarce. To address this issue, we retrospectively compared the long-term outcomes of patients with AA managed using two different dosing schedules at our center over the past 15 years.

## Materials and methods

The study included all patients with acquired severe or very severe AA aged > 12 years who were treated with hATG and cyclosporine at the Postgraduate Institute of Medical Education and Research (PGIMER, Chandigarh, India) between January 2003 and December 2021. This study was approved by our Institutional Ethics Committee. Informed consent was obtained from all adult subjects and the parents of patients aged less than 18 years.

AA diagnosis was based on a detailed history with clinical examination, complete blood counts, differential counts, peripheral blood smear, and bone marrow aspiration with biopsy. Severity was graded according to standard criteria [21]. Chromosomal breakage study (CBS) with diepoxybutane from peripheral blood lymphocytes was performed in all patients aged < 40 years. All patients underwent flow cytometric analysis of CD55 and CD59 to detect paroxysmal nocturnal hemoglobinuria (PNH) clones in granulocytes, monocytes, and RBCs. Baseline liver and renal function tests and serologies for Hepatitis B, C, and HIV were performed for all patients, and screening for Epstein–Barr virus, cytomegalovirus, parvovirus, and hepatitis A or E was performed wherever clinically indicated. We excluded patients who had undergone or were scheduled for allogeneic HSCT, had hypersensitivity to ATG or cyclosporine, had pre-existing renal failure, were diagnosed with inherited bone marrow failure syndrome (IBMFS), or had previously received any other formulation of ATG.

### Therapy

Two hATG formulations available in India were used: ATGAM (Pfizer Inc. NY, USA) and Thymogam (Bharat Serums and Vaccines, India). hATG at the standard dose (40 mg/kg/d × 4 days) or low dose (25 mg/kg/d × 4 days) was administered as an intravenous infusion over 6–8 h. The choice of dose and brand of hATG was predominantly based on patient affordability and the treating physician’s discretion; patients with limited finances received low-dose hATG. Methylprednisolone (2 mg/kg/day for 4 days, followed by a dose tapering over 2 weeks) was administered as prophylaxis against serum sickness. Cyclosporine was administered (5 mg/kg/day in two divided doses) from day 1 of hATG therapy and continued as per the response, with periodic monitoring of renal function and cyclosporine levels (target trough level, 150–250 ng/ml). Patients who experienced dose-limiting renal toxicity with cyclosporine were switched to mycophenolate mofetil (15 mg/kg/day in three divided doses). The patients were evaluated weekly for 2–3 months, monthly for 6 months after dose stabilization, and every 3 months thereafter. Cyclosporine was continued at a full therapeutic dose for the first 6 months. Subsequently, it was tapered or continued at a therapeutic dose depending on the hematologic response. After 2016, patients with AA without financial constraints also received eltrombopag (150 mg/day) for the first six months. hATG was considered a second-line therapy in patients who received it after more than 3 months of androgen treatment (danazol 200 mg three times a day) with or without cyclosporine. Both the groups received comparable supportive care; platelet transfusions were given to maintain platelet count ≥ 20 × 10^9^/L and PRBCs were transfused to maintain hemoglobin ≥ 7 g/dL. Febrile neutropenia episodes were managed as per departmental protocol.

### Criteria for response and relapse assessment

Response to IST was defined according to the standard criteria. Complete response (CR) was defined as hemoglobin > 10 g/dL, platelet count > 100 × 10^9^/L, and ANC > 1.0 × 10^9^/L in patients who did not receive transfusions [11]. Partial response (PR) was defined as transfusion independence (both for red blood cells and platelets), with a blood lineage not meeting the criteria for severe aplastic anemia, but insufficient for a complete response. No response (NR) was defined as persistently severe AA. The time to best response was defined as the time to attainment of stable PR or CR after IST administration. Relapse was defined as a decline in peripheral blood count to a level that met the criteria for severe or very severe aplastic anemia after the initial attainment of CR or PR [22]. In case of adequate response and improvement in hematological parameters, further bone marrow testing was not performed. This was repeated in cases of hematological deterioration, with suspicion of disease relapse or evolution to AML/MDS. The events included life-threatening bleeding, ICH, relapse, AML/MDS development, and death.

### Statistical analyses

The data were entered into an Excel spreadsheet and analyzed using SPSS software v.21 (IBM Inc., NY, USA). Continuous variables are expressed as mean and standard deviation or median and the interquartile range. Categorical variables are expressed as proportions. An independent t-test was used for normally distributed data, and the Mann–Whitney U test was used for non-normal data. The chi-squared test was used for categorical variables. Generalised estimating equation analysis was used to study the treatment effect over a time. Kaplan–Meier method was used to assess overall survival (OS) and event-free survival (EFS) and differences were compared using a log-rank test. Statistical significance was set at *P* < 0.05.

## Results

### Patient characteristics and treatment

**Fig. 1:**
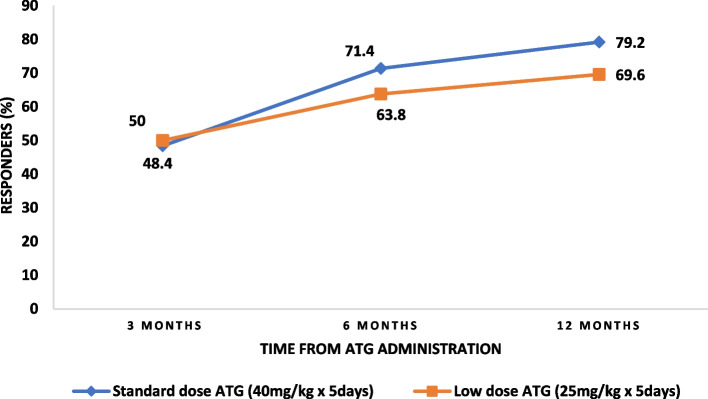
Response rate over a time across the group

Overall, 93 patients with AA who received hATG between January 2003 and December 2021 were included in the final analysis (Fig. 1). The mean age of the study population was 33.4 years (SD ± 15.3). Sixty patients (64.5%) were men. The majority of the patients had severe AA (SAA, 87 patients, 93.5%), six (6.4%) had very severe AA (VSAA). The baseline characteristics of the patients are summarized in Table 1. The median duration of symptoms before AA diagnosis was 3 months (range: 1–100 months), and the median follow-up duration after hATG administration was 52 months (range 3–229 months). One-third of patients (31/93, 33.3%) received standard-dose hATG, whereas the remaining two-thirds of patients (62/93, 66.7%) received low-dose hATG. The majority of the patients received hATG as first-line therapy (58/93, 62.4%). hATG formulation was ATGAM in 51 patients (54.8%) and Thymogam in 42 (45.2%) patients. Seventeen patients (18.2%) also received eltrombopag. The treatment characteristics are summarized in Table 2.

**Table 1:** Baseline characteristics of the study population

Variable	Total patients (*n* = 93)	Low dose hATG (*n* = 62)	Standard dose hATG (*n* = 31)	*P* value
Age, mean ± SD	33.4 ± 15.3	35.2 ± 15.5	29.7 ± 14.2	
Age ≤ 40 years n(%)	62 (66.7)	37 (59.7)	25 (80.6)	0.043
Age > 40 years n(%)	31 (33.3)	25 (40.3)	6 (19.4)	
Sex				
Male n(%)	60 (64.5)	40 (64.5)	20 (64.5)	1.000
Female n(%)	33 (35.5)	22 (35.5)	11 (35.5)	
Symptom to diagnosis interval in months, median (IQR)	3 (2,5)	3 (2,5)	3 (1.75, 5.25)	0.713
Severity of AA				
Severe AA n(%)	87 (93.5)	58 (93.5)	29 (93.5)	1.000
Very severe AA n(%)	6 (6.5)	4 (6.5)	2 (6.5)	
PNH clone				
Positive n(%)	33 (35.5)	24 (38.7)	9 (29.0)	0.133
Negative n(%)	41 (44.1)	29 (46.8)	12 (38.7)	
Not available n(%)	19 (20.4)	9 (14.5)	10 (32.3)	
Prior red blood cell transfusion n(%)	74 (79.6)	48 (77.4)	26 (83.9)	0.467
Prior platelet transfusion requirement n(%)	63 (67.7)	41 (66.1)	22 (71.0)	0.638
Hematologic parameters				
Hemoglobin (g/L), mean ± SD	6.4 ± 1.9	6.4 ± 1.9	6.3 ± 1.9	0.909
TLC (× 10^9^/L), mean ± SD	2.6 ± 1.2	2.5 ± 1.2	2.8 ± 0.9	0.268
ANC (× 10^9^/L), median (range)	0.6 (0.3–1.1)	0.6 (0.3–1.1)	0.7 (0.3–1.3)	0.481
ALC (× 10^9^/L), median (range)	1.5 ± 0.67	1.5 ± 0.64	1.8 ± 0.7	0.173
Platelet count (× 10^9^/L), median (range)	14 (8–2.2)	13 (9–20)	14 (7.8–23)	0.671

**Table 2:** Treatment characteristics of study population

Variable	Total patients (*n* = 93)	Low dose ATG (*n* = 62)	Standard dose ATG (*n* = 31)	*P* value
Diagnosis to ATG interval (months, range)	2 (1,5)	2 (1,5)	3 (1,6)	0.957
Timing of hATG				
As first-line, n(%)	58 (62.4)	37 (59.7)	21 (67.7)	0.449
As second-line, n(%)	35 (37.6)	25 (40.3)	10 (32.3)	
Eltromobpag with ATG				
Yes, n(%)	17 (18.3)	12 (19.4)	5 (16.1)	0.704
No, n(%)	76 (81.7)	50 (80.6)	26 (83.9)	
Formulation of ATG				
ATGAM	51 (54.8)	39 (62.9)	12 (38.7)	0.027
Thymogam	42 (45.2)	23 (37.1)	19 (61.3)	
Duration of IST after ATG (months), median (range)	25 (12,47)	25.5 (14.75,51.25)	25 (9,34)	0.072

### Response to therapy

Of the 93 patients, 72 (77.4%) showed a response to hATG during their follow up whereas 21 patients (22.6%) did not respond. The numbers of patients eligible for response assessment at 3, 6, and 12 months after hATG were 93, 86, and 80, respectively. The overall response to hATG at 3, 6, and 12 months was observed in 49.5%, 66.3%, and 72.5% of patients, respectively. Details of the responses to hATG are summarized in Table 3. There was no significant difference in ORR between the low and standard-dose groups at 3 months (31/62, 50%; vs. 15/31, 48.4%; *p* = 0.883), 6 months (37/58, 63.8%; vs. 20/28, 71.4%; *p* = 0.483), and 12 months (39/56, 69.6%; vs. 19/24, 79.2%; *p* = 0.382). GEE analysis revealed a comparable overall response between the low and standard-dose arms over time (Table 4, Fig. 1). The median (IQR) time to best response was 10 months (3–18 months), and the difference between the low-dose and standard-dose groups was not statistically significant [12 months (3–18 months) vs 6 months (3–18 months); *p* = 0.635]. The median duration of cyclosporine use after hATG was 25 months (range 3–144 months), with no significant difference between the two groups. A total of 23/93 (24.7%) patients who had partial or no response at earlier time points achieved CR after 12 months of hATG administration while continuing cyclosporine. The response rates did not vary significantly between the two hATG formulations (ATGAM and thymogam) in low and standard-dose groups (Supplementary Table 1).

**Table 3:** Hematologic responses of the study population based on hATG dose

Variable	Total patients (*n* = 93), n(%)	Low-dose ATG (*n* = 62), n(%)	Standard-dose ATG (*n* = 31), n(%)	*P* value
Overall response ( PR + CR)				
At 3 months	46/93 (49.5)	31/62 (50)	15/31 (48.4)	0.883
At 6 months	57/86 (66.3)	37/58 (63.8)	20/28 (71.4)	0.483
At 12 months	58/80 (72.5)	39/56 (69.6)	19/24 (79.2)	0.382
Response at 3 months				
CR	5/93 (5.4)	4/62 (6.5)	1/31 (3.2)	0.809
PR	41/93 (44.1)	27/62 (43.5)	14/31 (45.2)	
NR	47/93 (50.5)	31/62 (50.0)	16/31 (51.6)	
Response at 6 months				
CR	12/86 (14.0)	8/58 (13.8)	4/28 (14.3)	0.813
PR	45/86 (52.3)	29/58 (50.0)	16/28 (57.1)	
NR	29/86 (33.7)	21/58 (36.2)	8/28 (28.6)	
Response at 12 months				
CR	16/80 (20.0)	10/56 (17.9)	6/24 (25.0)	0.614
PR	42/80 (52.5)	29/56 (51.8)	13/24 (54.2)	
NR	22/80 (27.5)	17/56 (30.3)	5/24 (20.8)	
Best response				
CR	44/93 (47.3)	28/62 (45.2)	16/31 (51.6)	0.813
PR	28/93 (30.1)	19/62 (30.6)	9/31 (29.0)	
NR	21/93 (22.6)	15/62 (24.2)	6/31 (19.4)	
CR after 12 months	23/93 (24.7)	15/62 (24.2)	8/31 (25.8)	0.865
Time to best response (months), median(IQR)	10 (3–18)	12 (3–18)	6 (3–18)	0.635
Relapse on IST	11 (11.8)	7 (11.3)	4 (12.9)	1.000
Time to relapse (months), median (IQR)	44 (20–94.5)	51.56 (24.75–88.75)	35.0 (10–98)	0.330
Clonal evolution	2 (2.2)	0 (0)	2 (6.5)	0.109

**Table 4:** Generalized estimating equation analysis for respone to hATG over time

Variables	OR(95%CI)	*P* value
Treatment arm		
Standard dose ATG	1.28 (0.43–3.86)	0.659
Low dose ATG	1.00	
Time	1.89 (1.26–2.84)	0.002
Standard dose ATG x time	0.81 (0.50–1.30)	0.377
Low dose ATG x time	1.00	

**Fig. 2:**
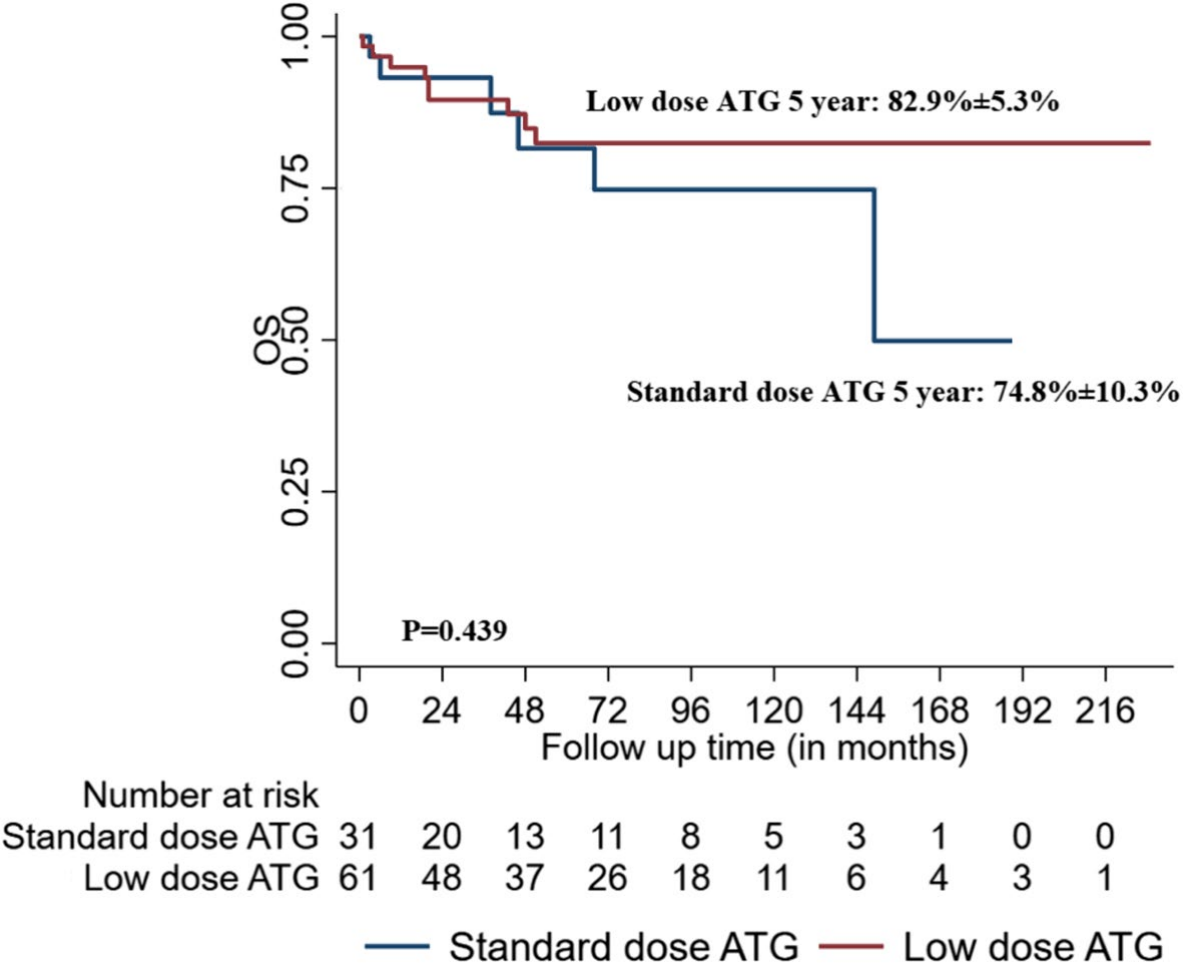
Kaplan–Meier analysis comparing overall survival (OS) between with low-dose and standard-dose hATG groups

**Fig. 3:**
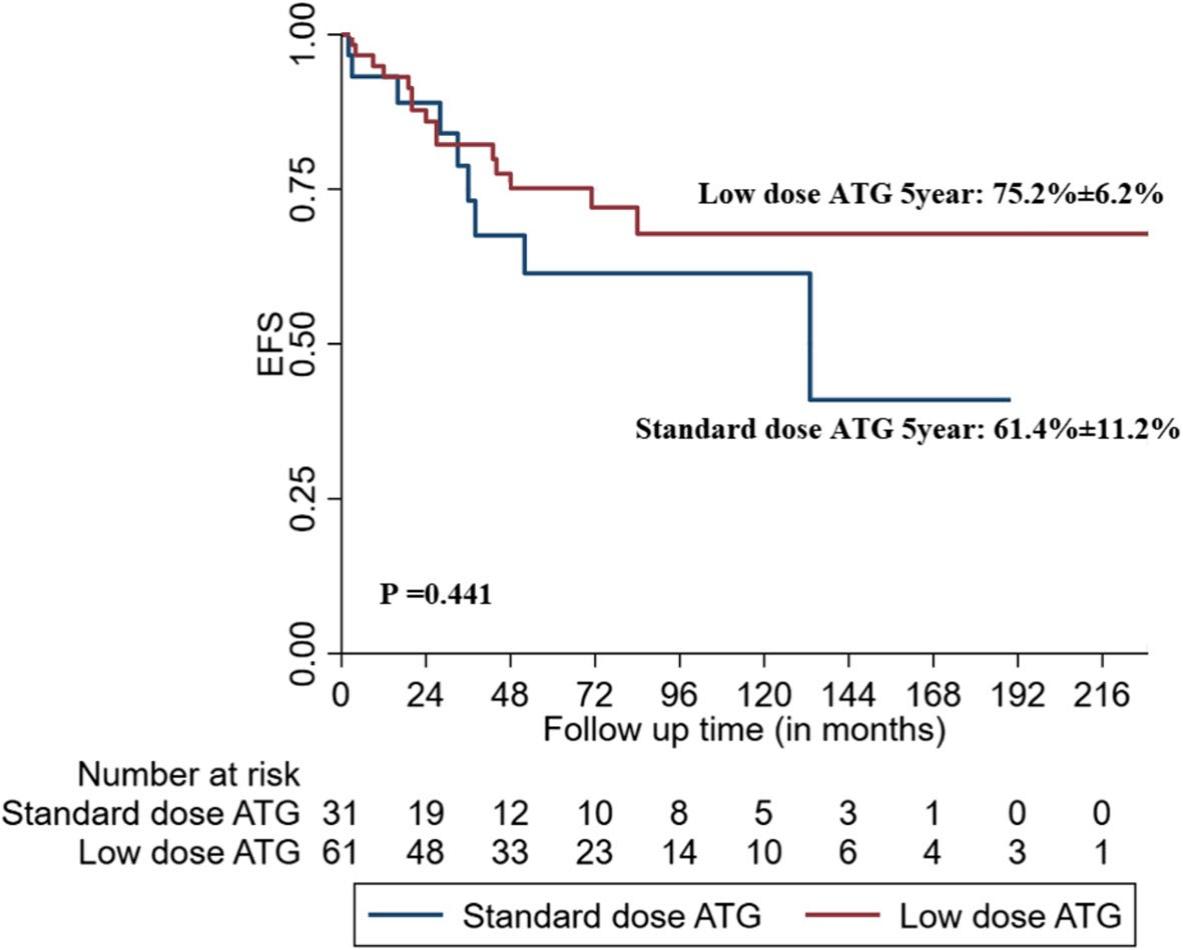
Kaplan–Meier analysis comparing event free survival (EFS) between with low-dose and standard-dose hATG groups

Of the 72 responders, 11 (11.8%) relapsed [7 (11.3%) in the low-dose group and 4 (12.9%) in the standard-dose group]. The median (IQR) time to relapse was 44 (20–94.5) months. Clonal evolution was observed in two patients (2.1%): one patient had acute myeloid leukemia with monosomy 7 at 83 months, and second patient had myelodysplastic syndrome with excess blasts-2 at 35 months (both in the standard-dose group). Further, 15 patients (16.1%) died during follow up. Most deaths occurred in the hATG non-responders (Supplementary Table 2). At the time of data analysis, 18 (19.3%) patients were lost to follow-up [10/62 (16.1%) in the LD group and 8/31(25.8%) in the SD groups]. The mean (standard deviation) 5-year Kaplan–Meier estimate of overall survival and event-free survival was 82.1 (4.6)% and 70.9 (5.5)% for the study population. The mean (standard deviation) 5-year Kaplan–Meier estimate of overall survival and event-free survival of those who received LD hATG dose versus SD hATG dose was 82.9 (5·3)% versus 74.8 (10·3)% (*P* = 0·439), and 75.2 (6.2)% versus 61.4 (11.2)% (*P* = 0·441) (Figs. 2 and 3).

## Discussion

IST with hATG has been the standard treatment option for transplant-ineligible AA patients for the past two decades. In this retrospective study on real-world outcomes of AA patients treated with hATG from 2003 to 2021, we found a 12-month response rate of around ~ 72% and 5 year OS/EFS which is similar to the reported outcomes from clinical trials and long-term registry studies [23–28]. Furthermore, the response and relapse rates in the standard and low-dose hATG groups at three, six, and 12 months were comparable. These comparable long-term outcomes of AA patients treated with the two dosing strategies suggest that the dose of hATG may be modulated depending on resource availability, without compromising efficacy.

The standard cumulative dose of ATGAM and its biosimilar, Thymogam, for the treatment of AA in combination with cyclosporine is 160 mg/kg; for lymphoglobulin, the dose is 75 mg/kg; and for thymoglobulin it is 17.5–18.75 mg/kg [29]. Supplementary Table 3 summarizes previous studies that have used alternative (other than standard) dosing strategies for various commercially available ATG formulations. A few other previous studies, including one from our center that used ATGAM or Thymogam hATG formulation, also yielded comparable outcomes with reduced and standard-dose regimens [19, 30, 31]. On the contrary, Kulagin et al. reported superior ORR with standard-dose hATG (~ 69%) when compared to low-dose (~ 51%) in a cohort of pediatric and adult patients with moderately severe or severe AA. However, in a later analysis by the same authors, multivariate analysis revealed no impact of the hATG dose on hematologic response [32, 33]. Various factors may account for heterogeneity in outcomes across studies using similar formulations and dosing strategies. In studies that included pediatric patients with AA, the probability of patients with occult inherited bone marrow failure (IBMF) syndrome receiving IST remains high [34]. There is increasing evidence to suggest that molecular testing with techniques like next-generation sequencing can reclassify up to 10–15% of acquired AA cases to an inherited bone marrow failure syndrome, particularly in pediatric cohorts and non-responders to IST [34, 35]. Moreover, outcomes vary according to AA severity and age of patients [36–38]. In our study, the mean age of the patient was 33 years, and only patients aged> 12 years with severe or very severe AA received hATG. Thus far, formal prospective dose-finding studies on hATG are lacking. The variable outcomes of hATG based on dose across retrospective studies, including ours, emphasizes the need for prospective dose-finding trials of hATG in the current era. As TPO receptor agonists have now become part of the standard therapy with hATG and CSA, the impact on the long-term outcomes of patients using different dosing schedules of hATG with the addition of TPO receptor agonists to oral cyclosporine needs to be determined.

Lack of response at 6 months after IST with hATG is the usual time point for defining hATG refractoriness and proceeding to alternative treatment options, such as rATG or allogeneic HSCT [39]. However, most patients in our study had limited prospects for allogeneic HSCT. Therefore, oral cyclosporine, with or without the addition of androgens, was continued at therapeutic doses beyond six months in most non-responders. Out of 29 non-responders at six months, nine patients (31%) died (Supplement Table 1). However, 7/29 (24.1%) attained CR on follow-up, while another 3 (10.3%) attained PR while continuing cyclosporine. Similar findings of delayed responses have been reported previously [24]. It is plausible that a large number of patients in the current cohort were possibly in a state of advanced stem cell exhaustion at the time of hATG administration, and therefore took a long time to attain a hematologic response.

The phenomenon of cyclosporine dependence is known to occur in up to 15–20% of patients treated with IST. The gradual tapering of oral IST may delay relapse [36, 40]. Considering limited prospects of second line treatments such as rATG or HSCT in the current cohort, a strategy of very gradual taper of CSA was followed in the current patient population. The median duration of oral cyclosporine administration following hATG was 25 months. Overall, 11 out of 72 responders (15.3%) relapsed after a median duration of 32 months. Out of a total of 11 relapses, seven relapsed after achieving PR as the best response and four relapsed from CR. The relapse rate in our study was comparable to that of most other contemporary studies in both the low- and standard-dose arms [10, 11].

The significant limitations of our study include its retrospective nature, small study population with missing data, lack of data on the incidence of infections, and lack of stringent monitoring of clonal evolution. The choice of a lower dose was based on previous experience rather than a specific dose-finding study. Over the past two decades, the diagnosis of IBMF syndrome and supportive care for patients with hematologic disorders have evolved significantly. This study spanned almost two decades, and the diagnostic workup for IBMF syndrome has evolved over this period. The use of more sensitive techniques to diagnose IBMFS and changes in supportive care may have affected outcomes. Only a small minority of patients in the study received eltrombopag; therefore, the effect of this drug with the two hATG dosing regimens could not be analyzed. Infections are the leading cause of morbidity and mortality in patients with AA [41]. The impact of the hATG dose on the spectrum of infections could not be specifically analyzed in the current study.

## Conclusion

In conclusion, we reported comparable long-term outcomes of AA using two different dosing schedules for hATG. Since the cost of IST with hATG in AA patients predominantly depends on the dose, transplant-ineligible AA patients living in LMICs may benefit from lower hATG dosing strategies. In the absence of alternative therapeutic options, the continuation of IST with cyclosporine beyond 6 months may benefit patients who have achieved only a partial or no response. Prospective dose-finding studies are necessary to determine the ideal dose of hATG for treating AA.

### Supplementary Information

**Additional file 1.**
