# Supplementary material for: Comparable outcomes with low-dose and standard-dose horse anti-thymocyte globulin in the treatment of severe aplastic anemia
Source: Blood Res. 2024 Feb 26;59(1):6. doi: 10.1007/s44313-024-00003-z (PMC10903521; doi:10.1007/s44313-024-00003-z)
Supplement: Supplementary file 1 — Additional file 1. [file 44313_2024_3_MOESM1_ESM.docx]

***Supplementary Table 1: Hematologic responses of the study population based on hATG formulation***

|  | **ATGAM;n(%)** | **Thymogam; n(%)** | **P value** |
| --- | --- | --- | --- |
| **Low dose ATG** | **n=39** | **n=23** |  |
| Overall Response* (PR+CR)  At 3 months  At 6 months  At 12 months | 19(48.7)  23(63.9)  27(75.0) | 12(52.2)  14(63.6)  12(60.0) | 0.790  0.981  0.242 |
| **Standard dose ATG** | **n=12** | **n=19** |  |
| Overall Response* (PR+CR)  At 3 months  At 6 months  At 12 months | 6(50)  8(72.7)  9(81.8) | 9(47.4)  12(70.6)  10(76.9) | 0.887  0.904  0.768 |
| **Standard and Low dose ATG (combined)** | **n=51** | **n=42** |  |
| Overall Response* (PR+CR)  At 3 months  At 6 months  At 12 months | 25(49)  31(66)  36(76.6) | 21(50)  26(66.7)  22(66.7) | 0.923  0.964  0.328 |

*Number of surviving patients on follow up at each time point were denominators for

calculation of response rates

***Supplementary Table 2: Details of deaths in study population***

|  | **Total patients**  **(n = 93)** | **Low dose ATG**  **(n = 62)** | **Standard dose ATG**  **(n = 31)** | **P value** |
| --- | --- | --- | --- | --- |
| Overall deaths after hATG  Total, n/N (%)  Among responders, n/N (%)  Among non-responders, n/N (%) | 15/93 (16.1)  5/72 (6.9)  10/21(47.6) | 9/62 (14.5)  2/47 (4.2)  7/15 (46.7) | 6/31 (19.4)  3/25 (12.0)  3/6 (50.0) | 0.55  0.22  0.89 |
| Deaths according to response at 3 months  Among responders, n/N (%)  Among non-responders, n/N (%) | 2/46 (4.3)  13/47 (27.6) | 0/31 (0)  9/31 (29.0) | 2/15 (13.3)  4/16 (25.0) | 0.04  0.77 |
| Deaths according to response at 6 months  Among responders, n/N (%)  Among non-responders, n/N (%) | 4/57 (7.0)  9/29 (31.0) | 1/37 (2.7)  7/21 (33.3) | 3/20 (14.3)  2/8 (25.0) | 0.09  0.66 |
| Deaths according to response at 12 months  Among responders, n/N (%)  Among non-responders, n/N (%) | 5/58 (8.6)  7/22 (31.8) | 2/39 (5.1)  6/17 (35.3) | 3/19 (15.8)  1/5 (20.0) | 0.17  0.52 |

| **Author, Year**  ***Supplementary Table 3. Key studies using alternative* dosing strategies of various ATG formulations for treatment of Aplastic Anemia*** | **Study**  **design** | **Antithymocyte Globulin(ATG) preparation** | **ATG Dose** | **n** | **Outcomes and Comments** |
| --- | --- | --- | --- | --- | --- |
| 1. Young et al  (1988) (1) | Randomized controlled  trial | ATGAM (Upjohn, Kalamazoo) | ATGAM 15mg/kg/d x 10 days (Total dose 150 mg/kg)  ATGAM 15mg/kg/d x 14 days followed by alternate day doses for 14 days  (Total dose 315 mg/kg) | 41  36 | Comparable outcomes between two groups at 3 months in acute severe aplastic anemia  ATG was administered without cyclosporine  No added benefit of higher cumulative dose of eATG |
| 2. Killick et al  (2006)(2) | Retrospective | Lymphoglobuline (Genzyme, Cambridge, USA) | Lymphoglobuline 5mg/kg x 5 days (Total 25 mg/kg) | 12 | 1/12 patients responded (Age >60 years) to low dose eATG  Median age of study population 71 years |
| 3.Atta et al  (2010)(3) | Retrospective | Lymphoglobulin (Lymphoglobuline®, Genzyme, Cambridge, MA, USA)  Thymoglobuline®, Genzyme, Cambridge, MA, USA | Lymphoglobulin 15 mg/kg/d x 5 days (Total 75 mg/kg  Thymoglobuline 2.5 mg/kg/d x 5 days (Total 12.5 mg/kg) | 42  29 | Overall response rate at 6 months was  59.5% for eATG (Lymphoglobulin)  and 34% for rATG (Thymoglobuline);similar response rates of rATG as compared to historical controls despite lower dose |
| 4. Yoshimi et al (2013)(4) | Retrospective | Lymphoglobulin (Lymphoglobuline®, Genzyme, Cambridge, USA)  Thymoglobuline®, (Genzyme, Cambridge, MA, USA) | Lymphoglobulin 15 mg/kg x 8 days (Total 120 mg/kg)  Thymoglobulin 3.75 mg/kg/d x 5 days (Total 18.75 mg/kg) | 96  32 | ORR at 6 months was 65% for eATG (Lymphoglobulin) and 34% for rATG (Thymoglobuline); no added benefit of higher cumulative dose of eATG as compared to other published studies on eATG |
| 5. Vallejo et al (2013)(5) | Retrospective | Thymoglobuline® (Genzyme, Cambridge, USA)  Lymphoglobuline (Genzyme, Cambridge, USA)  ATGAM (Upjohn, Kalamazoo) | Thymoglobuline dose range from 2.5 mg/kg – 4 mg/kg x 5 days (Total 12.5 – 20 mg/kg)  Lymphoglobulin 15 mg/kg/days x 5 days (Total 75 mg/kg)  ATGAM 40mg/kg x 4 days (Total 160 mg/kg) | 169  46  16 | ORR at 3 months was 73.2% for rATG (Thymoglobuline),75.4% for eATG (Lymphoglobulin and ATGAM combined)  Similar response rates as compared to historical controls of rATG despite lower cumulative dose used in a fraction of patients. |
| 6. Kulagin et al (2013)(6) | Retrospective | ATGAM (Pfizer, USA) | ATGAM 40 mg/kg x 4 days (Total 160mg/kg)  ATGAM 20 mg/kg x 5 days (Total 100mg/kg) | 74  81 | Overall response rate was 69% in higher dose eATG arm group and was 51% in lower dose arm(p = 0.023); results reported only in abstract form with limited follow up |
| 7.Kulagin  et al  (2014) (7) | Prospective | ATGAM (Pfizer, USA)  Thymoglobuline, Genzyme, Cambridge, MA, USA | ATGAM 40 mg/kg/d x 4 days(Total 160mg/kg)  ATGAM 20 mg/kg/d x 5 days (Total 100mg/kg)  Thymoglobuline 3.75mg/kg x 5 days | 55  52  18 | Overall response rate was 69% in higher dose eATG arm and 53% in lower dose eATG arm,  eATG dose had borderline significance for response on univariate analysis, dose was not significant predictor of response on multivariate analysis |
| 8.Scott et al  (2016)(8) | Retrospective | ATGAM (Pfizer, USA) | ATGAM 40 mg/kg/d x 4 days(Total 160mg/kg)  ATGAM 15mg/kg x 5 days(Total dose 75mg/kg) | 14  17 | Overall response rate (64 vs 71%, *P* = 1.0), relapse (33 vs 33%, *P* = 1.0), transformation (14 vs 24%, *P* = 0.66) infection (43 vs 47%, *P* = 1.0), overall and event free survival were comparable between standard and lower-dose cohorts. |
| 9. Malhotra et al  (2015)(9) | Retrospective | ATGAM(Pfizer,USA)  Thymogam(Bharat Serums and Vaccines,India) | ATGAM 25 mg/kg/d x 4 days(Total 100 mg/kg)  Thymogam 40mg/kg/d x 4 days(Total 160mg/kg) | 23  16 | Overall response rate was 77% at 6 months and was similar in lower dose ATGAM and standard dose Thymogam |
| 10.Diego V Cle et al  (2018)(10) | Retrospective | Lymphoglobuline (Genzyme, Cambridge, USA)  Thymoglobulin®, Genzyme, Cambridge, MA, USA) | Lymphoglobuline  Dose:30mg/kg/d x 5 days(total dose 150 mg)  Dose:15mg/kg/d x 5 days(Total dose 75 mg  Thymoglobuline  Dose:1-2.5mg/kg/day x 5 days  Dose:2.6-3.0mg/kg/day x 5 days  Dose:3.1-3.5mg/kg/day x 5 days  Dose:3.6-5.0mg/kg/day x 5 days | 85  56  29  46  38 | Overall response rate at 6 months was 59% and 31% for eATG and rATG respectively, dose-based responses for different eATG doses were not reported,dose of rATG did not influence response or survival |
| 11.Narita et al  (2019)(11) | Prospective randomized  trial | Thymoglobulin®, Genzyme, Cambridge, MA, USA) | Thymoglobuline  Dose:2.5mg/kg/d x 5 days(Total dose 12.5mg/kg)  Dose:3.5mg/kg/d x 5 days(Total dose 17.5mg/kg) | 112  110 | Overall response rate at 6 months was 49% for lower dose and 48% for higher dose rATG with no difference in efficacy and safety of low-dose versus higher-dose |
| 12. Gyi et al  (2020)(12) | Retrospective | Thymogam (Bharat Serums and Vaccines, India) | Thymogam 40-60 mg/kg  Thymogam 60-80 mg/kg  Thymogam 160 mg/kg | 9  5  5 | Overall response rate was 78.9% and response was attained even with 40mg/kg total dose |
| 12. Current study | Retrospective | ATGAM(Pfizer,USA)  Thymogam(Bharat Serums and Vaccines,India) | ATGAM  25mg/kg x 4 days(Total 100 mg/kg)  40 mg/kg x 4 days(Total 160mg/kg)  Thymogam  25mg/kg x 4 days(Total 100mg/kg)  40mg/kg/d x 4 days(Total 160mg/kg) | 39  12  23  19 | Overall response rate was 66.3% and 72.5% with no statistical difference in low dose and standard dose group, with comparable 10 year overall and event free survival |

*Standard cumulative dose for the treatment of AA in combination with cyclosporine is ATGAM: 160 mg/kg;Lymphoglobuline:75 mg/kg,Thymoglobuline:17.5 - 18.75 mg/kg; dosing strategies other than standard were termed as alternative dosing strategies

1. Young N, Griffith P, Brittain E, Elfenbein G, Gardner F, Huang A, et al. A multicenter trial of antithymocyte globulin in aplastic anemia and related diseases. Blood. 1988;72(6):1861-9.

2. Killick SB, Cavenagh JD, Davies JK, Marsh JC. Low dose antithymocyte globulin for the treatment of older patients with aplastic anaemia. Leuk Res. 2006;30(12):1517-20.

3. Atta EH, Dias DS, Marra VL, de Azevedo AM. Comparison between horse and rabbit antithymocyte globulin as first-line treatment for patients with severe aplastic anemia: a single-center retrospective study. Ann Hematol. 2010;89(9):851-9.

4. Yoshimi A, Niemeyer CM, Führer MM, Strahm B. Comparison of the efficacy of rabbit and horse antithymocyte globulin for the treatment of severe aplastic anemia in children. Blood. 2013;121(5):860-1.

5. Vallejo C, Montesinos P, Polo M, Cuevas B, Morado M, Rosell A, et al. Rabbit antithymocyte globulin versus horse antithymocyte globulin for treatment of acquired aplastic anemia: a retrospective analysis. Ann Hematol. 2015;94(6):947-54.

6. Kulagin A, Ivanova, M., Golubovskaya, I., Babenko, E., Kruchkova, I., Pronkina, N., Bond- arenko, S., Vavilov, V., Stancheva, N., Lisukov, I., Kozlov, V. & Afanasyev, B. . The horse ATG (ATGAM) total dose of 160 mg/kg as compared with 100 mg/kg is superior in achieving haematologic response in aplastic anaemia patients. . Bone Marrow Transplantation. 2013;48(2):S43.

7. Kulagin A, Lisukov I, Ivanova M, Golubovskaya I, Kruchkova I, Bondarenko S, et al. Prognostic value of paroxysmal nocturnal haemoglobinuria clone presence in aplastic anaemia patients treated with combined immunosuppression: results of two-centre prospective study. British Journal of Haematology. 2014;164(4):546-54.

8. Scott A, Morris K, Butler J, Mills AK, Kennedy GA. Treatment of aplastic anaemia with lower-dose anti-thymocyte globulin produces similar response rates and survival as per standard dose anti-thymocyte globulin schedules. Internal Medicine Journal. 2016;46(10):1198-203.

9. Malhotra P, Bodh V, Guru Murthy GS, Datta AK, Varma N, Varma S. Outcomes of immunosuppressant therapy with lower dose of antithymocyte globulin and cyclosporine in aplastic anemia. Hematology. 2015;20(4):239-44.

10. Clé DV, Atta EH, Dias DSP, Lima CBL, Bonduel M, Sciuccati G, et al. Rabbit antithymocyte globulin dose does not affect response or survival as first-line therapy for acquired aplastic anemia: a multicenter retrospective study. Ann Hematol. 2018;97(11):2039-46.

11. Narita A, Zhu X, Muramatsu H, Chen X, Guo Y, Yang W, et al. Prospective randomized trial comparing two doses of rabbit anti-thymocyte globulin in patients with severe aplastic anaemia. Br J Haematol. 2019;187(2):227-37.

12. Gyi AA, Htut KT, Han YN, Khine MM, Thandar HM, Thant YM. Outcome of Severe Aplastic Anemia Treated with Low Dose Immunosuppression in Resource Limited World during Pandemic: Single Center Experience from Myanmar. Blood. 2020;136(Supplement 1):27-.
